# Perspectives on the History and Epidemiology of the Varicella Virus Vaccine and Future Challenges

**DOI:** 10.3390/pathogens14080813

**Published:** 2025-08-16

**Authors:** Masayuki Nagasawa

**Affiliations:** Department of Pediatrics and Developmental Biology, Institute of Science Tokyo, 1-5-45 Bunkyo-ku, Tokyo 113-8519, Japan; mnagasawa.ped@tmd.ac.jp

**Keywords:** varicella zoster virus, chickenpox, shingles, attenuated live virus vaccine, Oka strain, COVID-19 pandemic, latent infection

## Abstract

The varicella attenuated virus vaccine, developed in Japan in the 1970s, has dramatically reduced the number of pediatric chickenpox cases over the past 30 years due to its widespread use. However, a small number of cases of chickenpox, shingles, aseptic meningitis, and acute retinal necrosis caused by vaccine strains have been reported. There are also issues that need to be addressed, such as breakthrough infections and the persistence of the preventive effect of vaccination. In addition, there is the possibility of the emergence of revertants or mutations in the vaccine strain. In recent years, subunit vaccines have been developed, their immune-stimulating effects have been demonstrated, and they are being applied clinically. In addition, development of an mRNA varicella vaccine is underway. In this review, the history and impact of the varicella vaccine are overviewed, as well as its future challenges.

## 1. Introduction

Chickenpox is an acute contagious disease caused by the varicella zoster virus (VZV). Until the end of the 19th century, chickenpox and smallpox were not clearly distinguished. In 1875, Steiner showed that chickenpox could be caused by inoculation with the blister contents of a chickenpox patient, and in 1888, von Bokay confirmed that a child susceptible to chickenpox could develop chickenpox from contact with a shingles patient. In 1954, Thomas Weller, Nobel Prize winner with John Enders and Frederick Robbins for the isolation of the poliovirus in 1954, confirmed that VZV could be isolated from the blisters of both chickenpox and shingles patients [1,2]. Varicella zoster virus is a DNA virus belonging to the alpha subfamily of the herpesviridae family, which latently infects sensory ganglia and reactivates to cause shingles. In the 1970s, the attenuated live varicella vaccine was developed for the first time in Japan, and its widespread use led to a dramatic decrease in the number of chickenpox cases in childhood.

A decrease in various virus infections has been reported during the COVID-19 pandemic due to social behavior restrictions, social distancing, and the promotion of non-pharmaceutical interventions (NPIs) [3,4,5]. On the other hand, recovery of virus infections varies depending on the biological characteristics of each virus and differs between regions and countries, probably due to differences in culture, lifestyle, and infection control mitigation policies by country or region [6].

**Figure 1:**
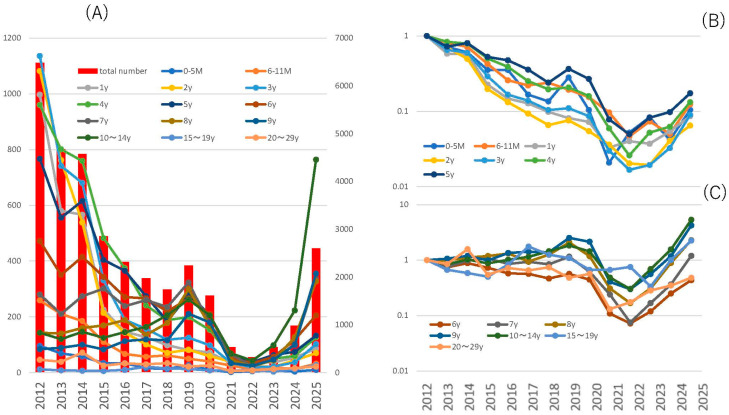
Outbreak trends of chickenpox patients according to fixed-point reporting in Tokyo, Japan. (**A**): Figure A shows the total number of reported chickenpox cases (bar graph; right axis) and the number of reported cases by age (line graph; left axis) from week 1 to week 21 of 2012–2025. (**B**,**C**): The relative number of reported outbreaks by age group is shown in the line graph, with the number of reported outbreaks in 2012 as 1.

A review of the trends in fixed-point outbreaks of chickenpox cases in Tokyo, Japan, since 2012 shows that although a prominent downward trend in outbreaks was observed after the introduction of routine two-dose varicella vaccination from 2014, a slight upward trend was observed from 2024 (Figure 1A). A look at trends by age shows that vaccination has a significant impact on children under 5 years of age, while children 6 years of age and older are almost completely unaffected, and the impact of the COVID-19 pandemic is observed across all age groups (Figure 1B,C) [7].

Against this background, this review will outline the history and impact of the varicella vaccine, as well as future challenges, especially focusing on childhood. In the text, the term “chickenpox” will be used to refer to disease caused by initial infection with the varicella zoster virus, “shingles” will be used to refer to disease with skin eruptions caused by reactivation after infection or attenuated live vaccination of varicella zoster virus, and “varicella” will be used to refer to infection with the varicella zoster virus in general.

## 2. Mechanism of Varicella Zoster Virus (VZV) Infection

Infectious VZV particles adsorbed on airway mucosal epithelium infect T cells or dendritic cells [8,9,10]. These cells are transported to regional lymph nodes, where virus production occurs and infected T cells are transported into the bloodstream to the rest of the body (primary viremia). Infected T cells infiltrate the dermis from blood vessels, migrate to the epithelium while producing virus, and infect epithelial cells with VZV [11]. Infected epithelial cells contribute to the formation of a bullous-pustular rash with virus production. Large amounts of infectious particles are contained within the rash and serve as a source of human-to-human transmission. During this process, infected epithelial cells simultaneously transmit the infection to uninfected T cells, resulting in systemic spread of the infection (secondary viremia). Through Langerhans cells or T cells, VZV eventually reaches the nerve fiber terminals and is transported retrogradely along the axon to the cell body, where it establishes a latent infection that lasts for life. VZV can reach ganglia during a viremia. VZV can also travel to ganglia by entering a sensory nerve directly at the site of a skin vesicle, from which the viral particle is transported within the neuron to the cell body in the dorsal root ganglion [12,13,14,15].

Glycoprotein B (gB), gE, gH, and gL are thought to be essential for VZV infection of cells [16]. However, the common receptors by which the VZV virus infects a variety of cells, including T cells, monocytes, dendritic cells, and epithelial cells, are not yet known. Cation-independent mannose-6-phosphate receptor (MPRci) has been reported to promote VZV infection [17], but it is not clear which molecules on VZV are associated with it. It was also reported that gE associates with insulin-degrading enzyme (IDE) and promotes VZV infection [18]. However, IDE and MPRci are not considered sufficient to be called entry receptors in VZV infection, since IDE and MPRci cannot trigger membrane fusion. On the other hand, as for latent infection of neurons, in the search for molecules with homology to paired immunoglobulin-like type 2 receptor α (PILRα)—which was identified as a cellular receptor for HSV-1 belonging to the same α-herpes subfamily—myelin-associated glycoprotein (MAG), which is specifically expressed in neurons, was identified [19]. By binding to gangliosides such as NgR1 (Nogo Receptor 1), NgR2, paired Ig-like receptor B (PIR-B), GD1a, and GT1b, MAG inhibits the growth of neuroaxons and is involved in the construction of neural tissue [20]. MAG was found to be an entry receptor for VZV that can associate with VZV gB and trigger membrane fusion [19].

## 3. Genes for Latent Infection

Molecular biological analysis has shown that transcripts such as open reading frame 4 (ORF4), ORF21, ORF29, ORF62, ORF63, and ORF66 are expressed in a latently infected state [21,22]. In a rat infection model, ORF4 deletion reduces the frequency of latent infection [23], but ORF21 is not essential for the establishment of latent infection [24]. In rats infected with VZV lacking ORF29, the establishment of latent infection in the trigeminal ganglion was significantly reduced, although the infection rate did not change, and the establishment of latent infection was also reduced when ORF29 was overexpressed, suggesting that the expression level of ORF29 regulates the establishment of latent infection [25]. ORF63 is not essential for growth in cell culture, but has been shown to contribute to the establishment of latent infection in rats [26].

Recently, a novel viral gene expressed in multiple human trigeminal ganglia was discovered and named VLT (Varicella Zoster Virus Latency-associated Transcript). In an experimental system using cultured cells, this VLT was found to specifically repress the expression of a gene called ORF61, which plays an important role in the very early stage of infection spread during VZV reactivation, suggesting that the VLT may work to maintain latent infection in the human body [27].

## 4. Immune Response to Varicella Zoster Virus (VZV)

From the moment of adsorption to the mucosal epithelium, VZV is exposed to the host immune response. First, the innate immune system triggers the activation of Natural Killer (NK) cells and the production of type I (alpha, beta) and type II (gamma) interferons (IFN). In vitro, both types of IFN inhibit VZV replication [28,29]. It is shown that IFN-α can relieve symptoms in immunocompromised children with chickenpox [30]. It is also reported that IL-6 is an important component of the innate immune response to varicella [31]. With the activation of innate immunity, the production of specific antibodies and T-cell immunity is also induced, and VZV-specific T cells are thought to be important in controlling viral replication both during early infection and during reactivation, especially during recovery after chickenpox onset [32,33]. Reactivation of VZV is associated with a decrease in immunity with aging, particularly with a decrease in cellular-mediated immunity (CMI) via VZV-specific T cells [34]. Because CMI can be stimulated by additional varicella vaccine immunization to suppress the onset of shingles [35], vaccination of the elderly has been recommended recently, as discussed later.

## 5. Attenuated Live Vaccine Against Varicella Zoster Virus (VZV)

The varicella vaccine strain was isolated in 1971 from a Japanese patient named Oka as the parent strain (wild strain). After 11 passages in human cells at the low temperature of 34 °C, 12 passages were made in fetal guinea pig cells to establish the vaccine strain (vaccine Oka strain: vOka-strain). Then, in 1974, Japan reported that the attenuated live vaccine was effective in preventing varicella infection in pediatric patients with secondary immunodeficiency [36]. Between vOka and wild-Oka strains, there are 42 nucleotide sequence substitutions and 20 amino acid substitutions, 8 of which are concentrated in the immediately early gene62 (IE62gene: the transactivator that is first initiated by intracellular growth) [37]. A recent deep sequencing study of distinct preparations of attenuated live varicella vaccine revealed 137 conserved core single-nucleotide polymorphisms, suggesting that attenuated live varicella vaccines are extremely stable during the vaccine manufacturing processes [38].

The mechanism of attenuated Oka vaccine strains (vOka strain) is still largely unknown. It is known that the wild strain blocks the production of Th1 cytokines IFN-γ and IL-12 by inhibiting the innate immune pathway by TLR2, thereby inhibiting the induction of Th1 by DC, while the vaccine strain rather promotes IL-12 production [39]. In terms of infection kinetics, the vaccine strain infects T cells and proliferates, as well as the wild type [8,40], but has been shown in the SCID-hu(skin) model to have reduced proliferative potential in the skin [41]. At the gene level, there are differences at 42 base sites between the parental and vaccine strains [37], and differential activation of viral genes by ORF62 protein (IE62) has been reported [42] and the presence of attenuated markers of proliferation in the skin in the region of ORF30-55 [43], suggesting that multiple gene products are likely to be involved in attenuated viral growth. Glycoprotein C (ORF14) has been shown to be important for the proliferation of VZV in skin cells, suggesting a link with the biological characteristics of vaccine strains [41].

## 6. Are Varicella Vaccinees Infectious?

A relatively large randomized controlled study was conducted in the early stage of vaccine introduction. For sibling examples, one patient was inoculated with the attenuated live vaccine and another with a placebo, and the results were observed. A total of 6 out of 493 (1%) in the placebo group showed increased antibody titer (infection) (no appearance of rash), and no rash was observed in the siblings of those with increased antibody titer. Based on the pattern of antibody titer elevation, three of the six patients may have been inoculated with an attenuated live vaccine by mistake. The possibility of “false positives” remains for the positive antibody titers. They concluded that the infectivity of the vaccinated is almost negligible [44].

On the other hand, eight cases (aged 4 months to 39 years) have been reported, who developed chickenpox after family members (two adults and four children) had a rash after varicella vaccination. From all eight patients with chickenpox, the vaccine strain was isolated. All vaccinees had rash appearance 12–24 days after vaccination, and infected patients had disease onset 16–21 days after rash appearance in vaccinees. None of the infected patients were immunocompromised, and all were mildly ill. Furthermore, five chickenpox cases (2 to 35 years old) infected from the vaccinees who developed shingles after 8 months to 2 years of vaccination have been reported. From all five chickenpox patients, the vaccine strain was isolated. Four of the infected patients were within the family, and one was within the school. All had mild rashes, and one had mild meningitis [45].

Varicella vaccination was given to 575 children with ALL (acute lymphocytic leukemia) who were in remission after treatment (10%) or during maintenance treatment (90%). In all patients under maintenance treatment, treatment was discontinued before one week and after two weeks of vaccination. Rash appeared in 40% of the patients, on average, 30 days (7–41 days) after vaccination. Twenty-one siblings (23%) of the ninety-three patients who had a rash after vaccination were infected. Sixteen siblings had rash after 14–22 days, and the vaccine strain was isolated from four siblings. Five patients were asymptomatic, but antibody titers increased [46]. Infectivity after vaccination with the attenuated live varicella vaccine is almost negligible, but if the vaccinees present with a rash or blistering rash after vaccination, they are considered to be infectious.

The results of a 10-year post-marketing surveillance indicate that adverse reactions to vaccination are few (<5%) and mild. Among the adverse reactions, skin rashes other than at the vaccination site were reported to include rash (4.2%), erythema (1.4%), and blistering (0.63%), with an estimated total frequency of skin rash of less than 0.5% [47].

## 7. Efficacy of Attenuated Live Varicella Vaccine in Preventing Chickenpox

A study in European countries reported that more than 90% of children under 10 years of age had chickenpox [48]. A survey conducted before vaccine dissemination in Japan reported that 96% had chickenpox by the age of 7 years [49].

A double-blind randomized study in children has shown that a single dose of attenuated live vaccine provides 90–100% varicella protection for several years [44,50]. The safety and efficacy of an attenuated live vaccine for uninfected adults was also confirmed, albeit on a small scale [51]. Based on the above results, the varicella attenuated live vaccine was approved in the United States in 1995. Between 1995 and 2006, the number of pediatric chickenpox cases decreased by 70%, hospitalizations for varicella decreased by 80%, and deaths from varicella decreased by 97% in the United States. However, antibody testing using the FAMA (Fluorescent Antibody to Membrane Antigen) assay showed that a single vaccination did not produce sufficient antibodies in 24% of cases [52]. In 2007, two-dose vaccination was recommended, and the number of chickenpox cases further decreased [53].

In Japan, which was the first country in the world to develop an attenuated live varicella vaccine, the attenuated live varicella vaccine was approved in 1986, and vaccination began the following year. For a while, voluntary inoculation was performed by self-pay, and one-dose inoculation was the mainstream. Subsequently, routine vaccination with two doses at public expense was introduced in 2014. A similar downward trend in chickenpox cases has been reported from Japan [54].

## 8. Childhood Shingles

A study of residents in Rochester, Minnesota, reviewing medical records from 1960 to 1981, reported a 2.8 to 20.9-fold higher incidence of shingles under 20 years of age in patients who had chickenpox within the first year of life [55]. It is considered that this is due to the fact that VZV spreads over a wide area of the body due to immature immunity, and that sufficient acquired immunity cannot be obtained in children under one year of age. Similarly, it has been reported that chickenpox incidence before the age of 1 year is a risk factor for childhood shingles [56,57]. Childhood shingles is less common than in adults and the elderly, mostly in secondary immunocompromised patients with childhood cancer or immunosuppressive drugs [58]. Before and after the introduction of the varicella vaccine, childhood shingles was reported to have decreased from 20–60/100,000 person-years to 14/100,000 person-years [55,59].

Fewer cases of shingles are reported with the varicella vaccine strain, with a 79% reduction in shingles in vaccinated individuals compared to natural infection [60]. A population-based active surveillance study in Antelope Valley, CA, USA, found a 55% reduction in the incidence of shingles among those under 10 years of age in 2006 compared to 2000. It also found that the incidence of shingles was 4 to 12 times lower in vaccinated individuals than in individuals infected with the wild varicella strain virus [61]. A single-center retrospective observational study in Japan also reported a decrease in shingles cases among healthy children from 5.0/year to 3.3/year between 1990 and 2000 and 2001–2017 after the introduction of the varicella vaccine [62]. The lower incidence of shingles in varicella vaccinees may be explained by the fact that only 50% of vaccinated children have a viremia, while almost 100% of children with chickenpox have a viremia, due to the lower proliferative capacity in the skin of the vaccine strain [63].

There is a difference in the site of onset (dermadrome) between shingles after chickenpox and shingles after vaccination. A report summarizes seven cases of childhood shingles in which the vaccine strain was identified, ranging in age from 1 year and 7 months to 3 years and 3 months, and one case had a history of chickenpox, as shown in Table 1. All cases had previously received one dose of the vaccine. The most common sites of shingles were in the cervical and lumbar areas [64]. This is markedly different from the incidence of shingles in adults and the elderly after chickenpox, which is reported to occur in 17.6% for head to face, 14.5% for neck to upper extremities, 31.2% for upper extremities to back of chest, 19.6% for abdominal back, and 17.2% for buttocks to lower extremities [65].

In the case of chickenpox, a viremia occurs in 100% of cases, the virus can spread throughout the body, and the virus migrates retrogradely from the sensory nerve fibers at the blister site to the sensory ganglia, where it causes latent infection. In the case of vaccination of the upper extremities, latent infection occurs in the cervical spine area, and in the case of vaccination of the thighs and buttocks, latent infection occurs in the lumbar spine area, resulting in the onset of shingles from these areas. In addition, reports from Japan [72] and the United States [73], which examined the incidence of shingles after vaccination of pediatric patients with leukemia, both found that the frequency of shingles was 10 times higher in cases in which a rash appeared after vaccination than in those in which it did not. On the other hand, studies from the United States [74], Europe [75], and Japan [76] both found that shingles after vaccination of healthy individuals tends to occur at a younger age, and the time between vaccination and shingles is shorter compared to the time between chickenpox and shingles. In cases where the rash appeared after vaccination, it is possible that sufficient immunity could not be acquired.

The incidence of postherpetic neuralgia is reported to be much less common in child-to-adolescent-onset shingles than in adult- or elderly-onset shingles [77].

## 9. Meningitis Due to Reactivation of Varicella Virus

A small number of cases of meningitis have been reported in healthy individuals with the varicella vaccine strain. Most cases of meningitis are associated with the onset of shingles. Cases of meningitis due to reactivation of the wild strain not presenting shingles have been reported [78,79]. There are only three case reports of vaccine strain meningitis due to reactivation without presenting shingles from a vaccinee [80,81,82]. 

A review of 12 cases of childhood varicella meningitis after shingles in which vaccine strains were identified is reported [83]. Eight cases had one vaccination (two had carcinoma, six were healthy), and four cases had two vaccinations (three were healthy). All cases developed meningitis after shingles (0.3 to 11 years after vaccination). Six cases presented shingles in the cervical area, one in the trigeminal area, and one in the lumbar area (infant immunized in the thigh). A report comparing childhood meningitis caused by vaccine strain reactivation versus wild strain reactivation found that vaccine strain cases were younger (7 ± 3.4 years vs. 11.9 ± 3.6 years, *p* = 0.0038) and were more related to shingles (100% vs. 55%, *p* = 0.04) [78]. In cases of chickenpox, the varicella virus latently infects the trigeminal ganglia due to the appearance of a varicella rash on the head and face and viremia. If reactivation occurs there, the infection may spread directly to the meninges even if shingles is not seen in the trigeminal area. Meningitis from shingles after vaccination is thought to progress from the sensory ganglia to the meninges via the meningeal cavity [83]. Table 2 shows a list of reports of VZV meningitis after the onset of shingles in which the vaccine strain was identified so far.

## 10. Acute Retinal Necrosis Caused by VZV Attenuated Live Vaccine

Acute retinal necrosis (ARN) is a rare but serious inflammatory condition of the retina, often caused by viral infections, primarily from the herpes family. It is defined as greater than one focus of retinal necrosis along with other criteria, such as retinal arterial involvement with occlusive vasculopathy, the progression of symptoms in the absence of active antiviral therapy, and finally a prominent inflammatory reaction in the anterior and vitreous chambers [97].

Immunocompetent patients also may develop this, but immunocompromised individuals are more prone to severe disease. Moreover, more cases of acute retinal necrosis due to herpes simplex 2 virus have been observed in young people. In contrast, cases due to herpes simplex 1 or VZV occur in the elderly [98]. There are two confirmed cases of ARN reported in which VZV vaccine strains were identified from the lesion site. Both of them were immunocompromised and 42 and 78 years old, respectively. Ocular symptoms appeared 11 weeks and 6 weeks after vaccination, respectively [99,100].

## 11. Maintenance of Immunity to VZV

**Figure 2:**
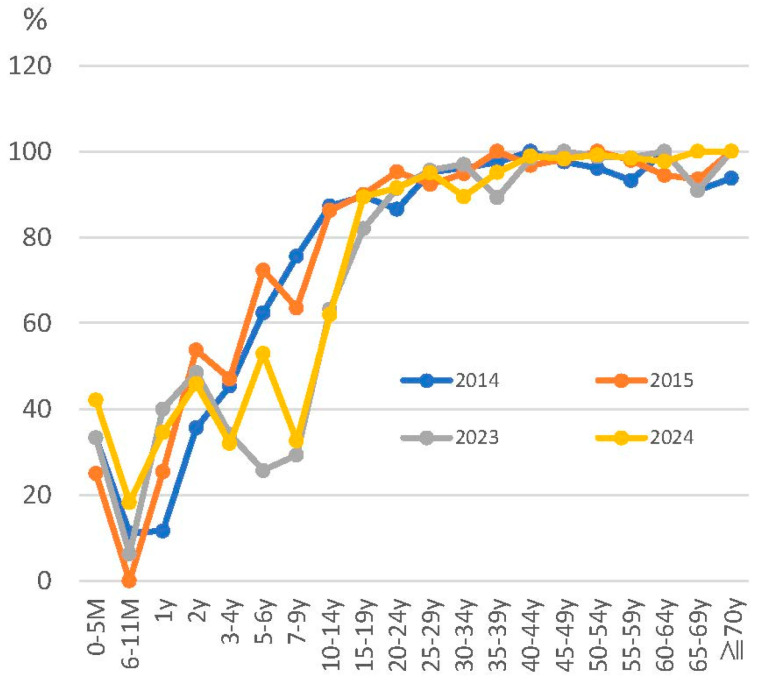
Trends in anti-varicella antibody titer (EIA ≥ 4) carriage rates by age in Japan are shown. The introduction of routine vaccination with two doses of varicella vaccine in Japan began in October 2014. Approximately 1400 people were tested for antibody titers each year. EIA: enzyme immunoassay. Data are available at https://id-info.jihs.go.jp/surveillance/nesvpd/graph/YearComparison/vzv2024/2024/20250602154218.html (available online 11 August 2025).

The National Institute of Health Risk Management in Japan has been investigating trends in varicella antibody titers by age as part of its “Survey on the Prediction of Infectious Disease Epidemics”. The graph of varicella antibody titers by age (2020–2024) (Figure 2) shows that more than half of the generation within a few years of receiving two varicella vaccines have EIA (enzyme immunoassay) antibody titers < 4 [101](antibody titers in children under 5 months are thought to indicate transferred antibodies from the mother). Referring to data on varicella vaccination coverage since 2016 from the public website [102], around 95% for one dose of the varicella vaccine and 90% for two doses of the varicella vaccine in Japan are maintained. In other words, more than half of infants have EIA antibody titers < 4 even after two doses of the varicella vaccine. In fact, trends in antibody titers show a transient increase in the ratio of EIA antibody titers > 4 at 2 years of age immediately after the two vaccinations. In Japan, two varicella vaccinations are given between the ages of 1 and 2 years.

Why is the increase in antibody titers not sufficient in infants even after two doses of vaccine, and why are antibody titers elevated in adults? It is antigen stimulation (i.e., exposure to the varicella virus) that drives the rise in antibody titers. There are two possible explanations for this: external exposure and internal exposure. Before the varicella vaccine was introduced, in environments where sufficient chickenpox cases were occurring, adults who already had varicella immunity would have had the opportunity to have their immunity stimulated by exposure to VZV in their daily lives. It has been shown that contact of a person with a history of varicella infection with a varicella patient stimulates immunity and increases immunocompetence [103]. However, when the varicella vaccine is introduced and the number of chickenpox cases in children declines by more than 90%, external exposure decreases dramatically. However, surveys of trends in anti-varicella antibody titers in adults show no significant change since 2014.

The other explanation is internal exposure (stimulation). Once the varicella zoster virus, a double-stranded DNA virus like the herpes virus of the same genus, has caused an infection, it latently infects ganglion cells and other organs in the body. Reactivation and proliferation in the skin along the nerves result in shingles. The internal stimulation theory suggests that even if shingles does not develop, mild reactivation may occur asymptomatically, which may provide antigenic stimulation and boost immunocompetence. This was first advocated by Hope-Simpson in 1952 [104]. Subsequently, searches for the virus in the saliva of astronauts returning from space travel have confirmed the reactivation of the varicella zoster virus asymptomatically [105]. In addition, the possibility of asymptomatic reactivation of varicella zoster virus in children and adults in general has been reported [106]. Another epidemiological study found no significant difference in the incidence of shingles between populations with no contact with children and those with contact with children [107]. This indicates that latent infection plays an important role in maintaining anti-varicella virus immunity over the long term.

As mentioned previously, the development of shingles due to reactivation of the varicella zoster virus is associated with a decrease in the function of varicella-specific T cells [34]. Concerns were raised that the introduction of the varicella vaccine would lead to an increase in shingles in adults and the elderly because of a drastic decrease in the number of chickenpox patients, which would reduce the external stimuli to maintain varicella-specific T cells [108]. While shingles in adults has continued to increase as chickenpox cases in children have decreased dramatically with the introduction of the varicella vaccine, the incidence of shingles has shown an increasing trend since the 1950s, long before the vaccine was introduced [109]. Multiple factors are thought to contribute to the increase in shingles, including increased stress in society, medically induced immunosuppression, an aging population, and increased diagnostic rates.

To reduce the increasing incidence of shingles, varicella-specific T cells must be stimulated and augmented [34]. Additional vaccination with an attenuated live vaccine has been shown to enhance varicella-specific T cells and contribute to the prevention of shingles development. Immunization with an attenuated live vaccine reduced the incidence of shingles by 51.3% (315/19,254 vs. 642/19,247) and postherpetic neuralgia by 66.5% (27/19,254 vs. 80/19,247) in persons over 50 years old [98,110,111].

If the patient is already immune through natural infection or attenuated live vaccination, a subunit vaccine has been shown to be effective enough to stimulate varicella-specific T cells. In a study of subjects aged 50 years and older, the subunit vaccination group showed an 89.8% reduction in incidence versus the placebo group over a mean 3.7-year observation period (23/6950 vs. 223/6950). There was also a 90.0% reduction in the 70–79 age group and an 89.1% reduction in the 80+ age group, independent of age [112,113]. It has attracted attention as an immune-boosting strategy that can be safely used in immunocompromised individuals because there is no risk of developing chickenpox or shingles due to the subunit vaccine compared to the attenuated live vaccine [114]. Following the subunit vaccine, an mRNA vaccine that attracted attention as a vaccine against SARS-CoV-2 has been developed as a varicella vaccine, and its efficacy in animal models has been reported [115].

## 12. Problems and Future Challenges with Varicella Attenuated Live Vaccine

### 12.1. Breakthrough Infection

Breakthrough infections were 3–5% per year over a 3-year observation period (431 patients aged 3–15 years). However, all of them were reported to have a milder disease than infection by wild strains [116]. A 7-year observational study found that annual breakthrough infections were 0.2–2.3%, cumulative breakthrough infections were less than 6.5%, and symptoms were all mild [117]. A report from Japan reported that in a follow-up study of 593 persons who received the attenuated live vaccine in 1987–1989, a cumulative total of 157/459 (34.2%) had breakthrough infections by 1996, all of which were mild cases [118]. In a 10-year observational study of approximately 2000 children aged 1 to 12 years, the authors found 94.4% protection with one dose of vaccine and 98.3% with two doses. It also states that antibodies were detected in all vaccinated individuals over a 9-year period [119].

### 12.2. Persistence of Immunity

Although various observational and epidemiological studies suggest that the vaccine’s efficacy in preventing varicella persists for 10–20 years or longer, there are variable factors, such as the boosting effect due to the prevalence of chickenpox, and some points are still unclear. With the widespread use of the attenuated live varicella vaccine, the number of wild-type chickenpox cases has been drastically decreasing, especially in developed countries. It may be necessary to examine and verify whether the current vaccine program is sufficient to prevent sudden outbreaks of wild-type varicella or novel mutated varicella virus.

### 12.3. Trends in Shingles Caused by Vaccine Strains

As mentioned previously, the incidence of shingles caused by vaccine strains has been reported to be lower than that of wild strains [60,61,109]. However, even in developed countries, only 30–40 years have passed since the vaccine was introduced. Advances in medicine and changes in social structure, such as an aging population, may lead to an increase in the number of people with secondary immunosuppressive conditions, which could significantly alter the population’s immunity situation. The long-term effects of vaccines are still unknown.

### 12.4. Emergence of Mutant Vaccine Strains

Quinlivan et al. compared the sequences of viruses isolated from skin eruptions caused by vaccine strains with those of vaccine strains and reported that several SNPs were found. However, these SNPs were not associated with increased viral virulence [120,121]. There have been no reports so far of reversion of vaccine strains to the parent strain or mutation to a highly virulent strain. However, monitoring by surveillance systems remains important.

## 13. Conclusions

The attenuated live varicella vaccine developed in Japan in the 1970s dramatically reduced the number of chickenpox cases in children over a period of about 30 years. It is regarded as a highly safe vaccine with very few adverse reactions. On the other hand, it is necessary to consider whether the current vaccine program for varicella infection is sufficient in an era when the number of vaccine strain infections exceeds the number of wild strain infections. Subunit and mRNA vaccines are promising new approaches to maintaining herd anti-varicella immunity. In addition, careful monitoring is needed to ensure that the possible emergence of varicella infections caused by newly mutated wild strains or vaccine strains does not pose a new threat.

## Figures and Tables

**Table 1:** Pediatric cases of shingles in children with identified and confirmed vaccine strains. This table is an edited and revised version of reference [64] by the author.

No	Age	Age at Vaccine	Varicella History	Interval	Region	Strain	Virus Isolation	References
1	1 y 7 m	1 y 3 m	yes (7 m)	4 m	L C6-7	vaccine	skin	*J. Am. Acad. Dermatol*. 1998; 38, 761–763 [66].
2	2 y 3 m	11 m	no	1 y 4 m	R C6-8	vaccine	skin	*Eur. J. Pediatr*. 2002; 161, 442–444 [67].
3	2 y 4 m	1 y 1 m	N/A	1 y 3 m	L chest-upper limb	vaccine	skin	*Pediatr. Infect. Dis. J*. 2008; 27, 847–848 [68].
4	2 y	1 y 5 m	no	7 m	L V1-2	vaccine	skin	*J. Clin. Virol*. 2016; 85, 44–47 [69]
5	3 y	1 y	N/A	2 y	R L2	vaccine	skin	*Pediatr. Dermatol*. 2017; 34, 665–667 [70].
6	2 y	1 y	no	1 y	L L4	vaccine	skin	*Pediatr. Dermatol*. 2017; 34, 665–668 [70].
7	3 y 3 m	1 y 8 m	no	1 y 7 m	L L4-S1	variant of vaccine	skin	*J. Child Neurol*. 2019; 34, 184–188 [71]

N/A = information was not available.

**Table 2:** Varicella meningitis pediatric cases in which the vaccine strain was identified and confirmed. All cases of meningitis presented secondary to shingles.

No	Age at 1st Vaccine	Age at 2nd Vaccine	Age at Meningitis	Site of Singles	Immunocompromised	References
1	1 y	none	1.3 y	Lumbar	yes	*J Infect Dis*. 2003; 188: 954–9 [84].
2	1.8 y	none	3 y	Trigeminal	no	*Pediatrics*. 2010; 125: e969–72 [85].
3	1.3 y	none	4 y	Cervical	no	*J Infect Dis*. 2008; 197 (Suppl 2): S170–7 [86].
4	2.4 y	none	4 y	Cervical	yes	*J Infect Dis*. 2008; 197 (Suppl 2): S170–7 [86].
5	1 y	none	7 y	Cervical	no	*Pediatr. Infect. Dis. J*. 2011; 30, 266–268 [87].
6	1 y	none	8 y	Cervical	no	*J Infect Dis*. 2008; 198: 1444–7 [88].
7	1 y	none	9 y	Cervical	no	*Ann Emerg Med*. 2009; 53: 792–5 [89].
8	1 y	none	12 y	Cervical	no	*J Infect Dis*. 2011; 203: 316–23 [90].
9	1.5 y	12 y	14 y	Thoracic	no	*J. Pediatr. Infect. Dis*. 2017; 12, 142–144 [91].
10	1 y	4 y	15 y	Lumbar	no	*Pediatrics* 2019; 144, e20191522 [92].
11	1 y	10 y	16 y	Thoracic	yes	*Pediatrics* 2019; 144, e20191522 [92].
12	1 y	5 y	14 y	Lumbar	no	*J. Child Neurol*. 2020; 35(13): 889-895 [93].
13	yes	yes	11 y	Trigeminal	no	*J Clin Microbiol*. 2023; 61(8): e0071422 [94].
14	1 y	1.5 y	12 y	Lumbar	no	*Emerg Infect Dis*. 2022; 28(7): 1523–1524 [95].
15	1 y	5 y	14 y	Yes (unknown)	no	*Viruses* 2021; 13(11):2287 [96].

## Data Availability

The raw data supporting the conclusions of this article will be made available by the authors on request.
